# Editorial: Mind the Sustainable Food: New Insights in Food Psychology

**DOI:** 10.3389/fpsyg.2021.725579

**Published:** 2021-09-20

**Authors:** Valentina Carfora, Gianni Cicia, Mark Conner

**Affiliations:** ^1^Department of Psychology, Catholic University of the Sacred Heart, Milan, Italy; ^2^Department of Agricultural Sciences, University of Naples Federico II, Portici, Italy; ^3^School of Psychology, University of Leeds, Leeds, United Kingdom

**Keywords:** food psychology, sustainability, health, environment, food choice

## Sustainable Food Psychology: An Emerging Area

As the prime connexion between people and the planet, the history of food is as long as that of mankind. Food choices and whole diets have long been influenced by environmental, psychological, sociocultural, and technological factors. Among the various disciplines studying how food choices are closely connected to the biological and sociocultural evolution of humanity, Food Psychology applies psychological theories, and methodology to consider the relationships among food attributes, consumers' characteristics, and sociocultural influences.

Recognising the key role of dietary habits in health, for many decades Food Psychology mainly focused on understanding and promoting healthy eating habits. However, in recent years, a growing focus has been on sustainable food choices. Such sustainable food choices encompass both benefits to human health and protection of the environmental (EEA (European Environment Agency), 2017; FAO, 2018) and was the definition of sustainable food we used here. For example, food choices or whole diets that reduce or even eliminate meat from the diet would meet this definition of a sustainable food choice (e.g., Chai et al., 2019). As Schmidt and Mouristen propose in the present research topic, lacto-ovo-vegetarian or flexitarian diets might allow people to change their dietary habits without replacing food with nutritional supplements, changing fundamental social and ethnic traditions, or being exposed to cravings for umami.

Although researchers have an increasingly good knowledge of the impact of food consumption both on health and the environment, the general public mostly have a relatively poor understanding of what constitutes a sustainable diet. Moreover, understanding sustainable food choices—and even more convincing people to adopt them—still faces many barriers. Among these barriers are those related to complex attributes of the food products, social and psychological limitations, and the small number of ways in which researchers and policy makers can promote sustainable food choices (Cheah et al., 2020; Smiglak-Krajewska and Wojciechowska-Solis, 2021).

## Influences of Food Attributes on Sustainable Food Choices

In relation to how food attributes affect consumers' choices, one interesting recent debate is related to the influence of food reputation and country of origin. To considered how the impact of food reputation on food preferences varies across cultural contexts, in the present research topic, De Dominicis et al. validate three context-specific versions of the Food Reputation Map (FRM) to measure food reputation across twenty-three specific indicators, further grouped into six synthetic indicators of food reputation in Italian, English, and Chinese populations. More widespread use of the FRM could further our understanding in relation to sustainable food choices. In addition, Bonaiuto et al. analyse the effect of country of origin by identifying a social-psychological profile of “Italian Sounding” products—i.e., the “Italian appearance” of food products irrespective of its country of origin—when compared to both “Made in Italy” products and “Generic Foreign” products across three different countries (Italy, China and USA). Moreover, they show how food reputation mediates the impact of the perception of food as Italian on consumers' willingness to purchase it.

Relatedly, an emerging line of research concerns how new categories of foods, such as so-called “sustainable food alternatives,” are perceived by consumers and can facilitate the pursuit of more sustainable diets. In the present special issue, Chriki et al.'s systematic review examines cultured meat as an alternative for consumers who want to be more ethically minded but do not wish to avoid meat altogether. Their review shows that researchers focused initially only on technical aspects of artificial meat, while more recently they took into account how consumers' beliefs (e.g., health value and product acceptance) determine its purchase. Furthermore, in an opinion article, Mouritsen and Schmidt discuss how to increase interest in consumption of seaweed and cephalopods by considering both psychosocial factors preventing one from eating them, and how to generate a more positive image by proposing different words to connotate them.

## Effects of Psychosocial Determinants on Sustainable Food Choices

Concerning how consumers' beliefs affect their sustainable food choices, Food Psychology could usefully focus on several key determinants, such as cognitive, moral, affective, and personality dimensions. An emerging topic in relation to the cognitive influences related to food choice relates to people's trust toward food production. In this regard, Canova et al. highlight the importance of people's trust in organic products as a meaningful antecedent that boosts the psychosocial processes that are assumed to underlie both purchasing intentions and choice behaviours. Recent studies in this area are also investigating the implications of moral motives related to the choice of sustainable food. For example, Lai et al. investigate the different implications of moral and non-moral motivators for reducing meat intake. Their two studies show the direct impact of health concern and the indirect role of “biospheric values” and descriptive norm (via personal norm). In relation to affective influences on sustainable food choice, Papadakis et al. examined how negative affect influences the relationship between environmental cues to high-calorie snacking and snacking behaviour. Finally, Dijksterhuis et al. draw upon psychological personality theory and propose unique underlying factors that can distinguish among consumers' personalities. They also explore the relationship between the identified factors and consumers' preferences for receiving certain forms of dietary advice/information.

Importantly, even when food products have sustainable attributes and consumers have positive attitude and good intentions toward eating sustainably, often people still do not select sustainable foods or diets (de Ridder et al., 2017). A sustainability gap. Research is necessary to bridge this gap between favourable values, attitudes and actual consumption of more sustainable food products and diets. In this regards, Schäufele and Janssen analysed the value-attitude-behaviour relationship and found that different types of food purchase is driven by the same food-related values but their relative importance differs based on category of product. Starting from the urgent need to reduce this gap, Vermeir et al. propose a comprehensive theoretical framework for future research on this topic, and highlight behavioural solutions for environmental challenges in the food domain from an interdisciplinary perspective.

## Interventions Aimed at Increasing Sustainable Food Choices

The above research could form the basis of interventions aimed at increasing sustainable eating. The review by Abrahamse indicates that interventions targeting sustainable food choices can rely on both unconscious or automatic decision-making processes (e.g., nudging), and more deliberative decision-making (e.g., information provision). Relatedly, for interventions leveraging less deliberative decision-making processes, Dijkstra and Elbert evaluated whether inducing voluntary eye movements during the processing of the auditory persuasive information prevented defensiveness and thereby increased the effectiveness of messages aimed to increase fruit and vegetable consumption. In relation to how to promote sustainable food choices via stimulating a deliberative decision-making process, the effectiveness of the information provision can be enhanced by manipulating message content and/or message framing. As an example of how to differentiate the content of the messages, Wolstenholme et al. showed that the nature of the health and/or environmental information provided were effective in reducing red and processed meat consumption compared to a no message control group, with some effects remaining 1-month later. Leveraging the framing of the messages, Carfora et al. found that gain and non-loss messages activated an integrated emotional and cognitive processing of the health recommendation, while loss and non-gain messages mainly activated emotional shortcuts toward attitude and intention.

## Future Directions: Where Shall We Go?

To sum up, the present research topic focused on sustainable food and dietary choices that can promote both health and environmental gains. The included papers all address directly how their work can enhance theory, methodology, and communication strategy, which could accelerate advances in the field of sustainable Food Psychology. Overall, the papers highlight the need to examine various possible moderators and mediators of effects on food choice and also the need to assess long-term effects. Initiating sustainable food choices and diets will have little impact on individual's health or on protecting the environment. It is only long-term changes that will have these effects. We would highlight this as an important and less studied aspect of sustainable food—these changes to food choices and whole diets need to be taken up by large portions of the population and over prolonged periods of time if the population health and environmental benefits are to be realised. It is also worth noting that the methodologies and approaches taken in the papers included in the special issue are heterogeneous. This is perhaps because of the lack of a common conceptual framework to drive work on understanding and promoting sustainable food choices and diet in this area. For example, most of the included papers focused on different types of food choice (e.g., meat reduction or snacking) rather than considering the more difficult question of adherence to a sustainable whole diet, which is key to obtaining health and environmental benefits (de Ridder et al., 2017). Future research and theorising that attempts a more holistic assessment and integration of the multitude of factors that influence food choices would be particularly valuable. Such integration might also usefully focus on the sensory (taste, smell, appearance, and texture of food) and environmental factors (such as salience of food and distractions), aspects that are too often overlooked in the field of sustainable food choice. Finally, future research might also identify the extent to which research on sustainable foods can draw upon existing work on healthy food choices and related area or needs to develop in new directions.

## Author Contributions

VC conceived the idea of this editorial. All authors contributed to the Editorial revision, read and approved the submitted version.

## Conflict of Interest

The authors declare that the research was conducted in the absence of any commercial or financial relationships that could be construed as a potential conflict of interest.

## Publisher's Note

All claims expressed in this article are solely those of the authors and do not necessarily represent those of their affiliated organizations, or those of the publisher, the editors and the reviewers. Any product that may be evaluated in this article, or claim that may be made by its manufacturer, is not guaranteed or endorsed by the publisher.

## References

[B1] Chai B. C. van der Voort J. R. Grofelnik K. Eliasdottir H. G. Klöss I. Perez-Cueto F. J. A. (2019). Which diet has the least environmental impact on our planet? A systematic review of vegan, vegetarian and omnivorous diets. Sustainability 11:4110. 10.3390/su11154110

[B2] Cheah I. Shimul A. S. Liang J. Phau I. (2020). Drivers and barriers toward reducing meat consumption. Appetite 149:104636. 10.1016/j.appet.2020.104636 32097692

[B3] de Ridder D. Kroese F. Evers C. Adriaanse M. Gillebaart M. (2017). Healthy diet: health impact, prevalence, correlates, and interventions. Psychol. Health 32, 907–941. 10.1080/08870446.2017.1316849 28447854

[B4] EEA (European Environment Agency) (2017). Food in a Green Light: A Systems Approach to Sustainable Food. Available online at: https://www.eea.europa.eu/publications/food-in-a-green-light (accessed July 9, 2021).

[B5] FAO (2018). Transforming Food and Agriculture to Achieve the SDGs: 20 Interconnected Actions to Guide Decision-Makers. Rome: FAO. Available online at: http://www.fao.org/3/I9900EN/i9900en.pdf (accessed July 9, 2021).

[B6] Smiglak-Krajewska M. Wojciechowska-Solis J. (2021). Consumption preferences of pulses in the diet of polish people: motives and barriers to replace animal protein with vegetable protein. Nutrients 13:454. 10.3390/nu13020454 33573021 PMC7912341

